# “I’m Doing the Best that I Can”: Mothers Lived Experience with Food Insecurity, Coping Strategies, and Mental Health Implications

**DOI:** 10.1016/j.cdnut.2024.102136

**Published:** 2024-03-21

**Authors:** Rachel A Liebe, Kathleen J Porter, Leah M Adams, Valisa E Hedrick, Elena L Serrano, Natalie Cook, Sarah A Misyak

**Affiliations:** 1 Department of Nutritional Sciences, Oklahoma State University, Stillwater, OK, United States; 2 Department of Human Nutrition, Foods and Exercise, Virginia Tech, Blacksburg, VA, United States; 3 Department of Public Health Sciences, University of Virginia, Christiansburg, VA, United States; 4 Department of Psychology, George Mason University, Fairfax, VA, United States; 5 The Virginia Cooperative Extension Family Nutrition Program, Department of Human Nutrition, Foods and Exercise, Virginia Tech, Blacksburg, VA, United States; 6 Department of Population Health Science, Virginia Tech, Blacksburg, VA, United States

**Keywords:** food insecurity, maternal health, mental health, social stigma, mothers

## Abstract

**Background:**

Food insecurity can have lasting physical and mental health consequences. The experience of food insecurity within a household may disproportionately impact mothers because they tend to manage the household food environment.

**Objective:**

This study sought to understand the stresses faced by United States mothers experiencing food insecurity, related coping mechanisms, and the impacts of these stressors on their mental health.

**Methods:**

Semistructured interviews were conducted in May and June 2022 with a purposive sample of Virginia mothers who reported experiences of food insecurity. Participants were recruited from a related survey and university and community LISTSERVs. Interviews were transcribed and analyzed by trained coders. A thematic analysis was conducted to describe themes that emerged from the data. Virtual interviews were 20–60 min in duration. Mothers with children living in their household, having experienced food insecurity, and living in Virginia were eligible.

**Results:**

The following 3 themes emerged from the interviews with the mothers (*n* = 15): *1*) food insecurity added stress to mothers’ lives in multiple ways (e.g. worry about obtaining the “right” foods and internalized or experienced stigma), *2*) mothers used positive and negative coping strategies to address the impacts of these stressors (e.g. use of community resources and reduced personal food intake), and *3*) the stressors and coping strategies had varying impacts on mothers’ mental health (e.g. added to existing mental health challenges or reduced their mental capacity to make changes).

**Conclusions:**

Study findings suggest that a multilevel and tailored approach to address diverse stressors is warranted. Future research should explore emotional coping strategies that comprehensively empower mothers to manage stressors, leverage resources, and reduce social stigma associated with food insecurity and accessing nutrition and mental health assistance. This may improve their household food security and mitigate the burden of stressors on their mental health because system-level solutions to food insecurity are pursued.

## Introduction

For a household to be considered food secure, safe, and nutritious foods must be consistently accessible in socially acceptable ways [1]. Food insecurity, which is a public health crisis [2], disproportionately affects female caregivers (referred to collectively as mothers for the remainder of the manuscript) with incomes below the United States federal poverty level. Although 12.8% of all United States households experienced food insecurity in 2022, 36.7% of households with an income below the federal poverty level experienced food insecurity. Additionally, 17.3% of households with children and nearly 33.1% of households headed by a single mother experienced food insecurity [3]. The experience of food insecurity may be different for mothers than for other household members. Mothers tend to be responsible for household food decisions and may select and implement behavioral coping strategies to manage limited food resources [4]. Additionally, mothers often shield children from experiencing food insecurity, often by limiting their own food intake [4].

There is growing evidence suggesting an association between food security and mental health [[4], [5], [6], [7], [8], [9], [10], [11], [12], [13]]. Although the directionality of that relationship is still being explored, there is evidence to suggest food insecurity impacts mental health [4,6,13]. Mental health differs from mental illness, which is a diagnosed health condition, and encompasses emotional, social, and psychological health and well-being [14]. For mothers, food insecurity is associated with greater stress, symptoms of anxiety and depression, and prevalence of a diagnosed mental illness, than those not experiencing food insecurity [6,13,15,16]. The effect of food insecurity on maternal mental health has been associated with increased depression and anxiety among children in the household [7,[17], [18], [19]].

In the United States, the relationship between food security and mental health is impacted by factors including physical health, social support, and behavioral food coping strategies [4,[6], [7], [8],^12^,^13^,^20^,^21^]. Social support and behavioral food coping strategies have been shown to mediate the relationship between food insecurity and mental health outcomes in different ways [5]. Although there is limited evidence on directionality, there is evidence to suggest social support may reduce some of the negative mental health effects associated with experiencing food insecurity [10,[22], [23], [24]]. Anxiety and depressive symptoms have been negatively correlated with physical health and social support [6,10,20,22,[25], [26], [27], [28], [29], [30], [31]]. In contrast, the usage of more behavioral food coping strategies is associated with being more food insecure and greater symptoms of mental illness [5,6].

Behavioral food coping strategies can be used in response to a stressor (i.e. an event causing stress). Common stressors among people at risk of food insecurity can be both chronic and acute, including loss or reduction of employment, emergencies or natural disasters, and unexpected illness or death of a household member [32]. There are many behavioral food coping strategies that are used by people experiencing food insecurity. Common strategies to obtain food include participating in nutrition assistance programs, couponing, purchasing items on sale or substituting cheaper items, and visiting food pantries [21,33]. People may also leverage strategies to stretch existing food resources such as reducing portion sizes or skipping meals, especially at the end of the month when assistance benefits run out [21,34]. Strategies may also be not directly food related such as reducing spending in other categories [33].

Coping strategies can be categorized as task-, emotion-, or avoidance-oriented based on the categorization of coping strategies commonly used in psychology [35]. Task-oriented strategies seek to identify a solution to the specific stressor (e.g., seeking improvement in circumstances), emotion-oriented strategies seek to regulate the emotional response (e.g. talking with friends about the experience), and avoidance-oriented strategies seek to forget or hide stressors (e.g. hiding the experience from friends). All categories of strategies can have positive or negative effects on mental health [29]. The type of strategies people rely on and the effect on mental health are highly individual and vary by contextual factors and available resources, but having to rely on more strategies is associated with worse mental health [5,6,35]. Previous research suggests that food insecurity may constrain available coping resources, such as social support, leading to chronic stress and detrimental effects on mental health [36]. There is a documented relationship between insufficient coping, chronic stress, and poor mental health outcomes [[36], [37], [38], [39], [40]].

Despite an understanding of the potential effects of the relationship between food security and maternal mental health, there is limited qualitative evidence to explore mothers’ perceptions of how stressors and coping strategies contribute to mental health outcomes that impact their daily lives. A qualitative approach is useful for initial exploration of complex phenomena and for providing rich descriptions that may not be thoroughly captured through quantitative methods [41]. The purpose of this study was to understand the perspective of Virginia mothers experiencing food insecurity on the stressors they face, the coping strategies they utilize, and the implications for their mental health. A greater understanding of mothers’ experiences is critical for the development of interventions that improve maternal mental health for those experiencing food insecurity.

## Methods

This descriptive qualitative study used semistructured interviews and was part of a larger study with an explanatory sequential mixed methods design aiming to develop a conceptual framework of the relationship between food security and mental health. This study was reviewed and approved by Virginia Tech Institutional Review Board. Participants received a $25 gift card for their time. This study was impacted by the residual economic effects of the COVID-19 pandemic shutdowns on inflation in the prices of both food and gas and the formula shortage that likely impacted participants [42,43].

### Participant recruitment

We used convenience sampling to identify mothers for this study. Mothers were recruited between May and June 2022. Potential participants either expressed interest in a follow-up interview after completing a survey about food security and mental health that targeted mothers in Virginia living at or <100% of the federal poverty level [6] or via emails to university and community organization LISTSERVs in Virginia. Information about the study was provided in the email through a flyer. Interested participants emailed study staff to express interest and were sent a short screener survey to complete. Eligibility criteria included identifying as a woman, having children living in the household, being >18 y old, having recent or current experience of food insecurity, and living in Virginia. Participants were asked to identify their gender and report yes or no to having children in the household on the short survey. Because most participants had previously taken the survey described above with similar eligibility criteria, living in Virginia and being >18 y old was confirmed prior to the start of the interview. All participants met both eligibility criteria. Eligible participants were contacted by email to confirm their interest, explain the study’s purpose, and schedule a virtual interview [via Zoom on a computer or telephone] at a time convenient for them. Because experiences of food insecurity are often episodic [44], participants were asked during the interview if they had recent experiences (past 12 mo) of food insecurity. A participant (*n* = 1) who indicated no recent experience was excluded from the analysis.

### Measures

Two measures were used in this study: a short survey and an interview protocol. The survey, which took ∼10–15 min to complete, assessed current (past 30 d) food security status using the USDA Household Food Security Module 6-item short form and mental health using the Patient Reported Outcome Measures Information System (PROMIS) Global Mental 2a Scale v1.2 [45,46]. The validated, 6-item short form of the USDA Household Food Security Module [39] was scored according to the Guide to Measuring Household Food Security. Participants’ food security status was then categorized as high/marginal (scores 0 and 1), low (scores 2–4), or very low (scores 5 and 6) [45]. Raw scores on the 2-item PROMIS scale were converted to T-scores standardized to the United States population with an average score of 50 and a 10-point SD. A score >50 indicated better mental health and <50 indicated worse mental health than the average American. T-scores can range from 25.8–64.6. Participants were also asked to identify their race and ethnicity on the survey.

An interview guide (Supplemental Table 1) was developed specifically for this study to explore constructs identified during a cross-sectional study of exploring the relationship between food security and mental health among food-insecure mothers in Virginia [6]. These constructs include social support, behavioral food coping strategies, and assistance resources. To tap into these constructs, the interview guide assessed the following 4 domains: *1*) usual family food experience, *2*) current/past experiences of food insecurity, *3*) effects of food insecurity on mothers’ mental health, and *4*) needed resources to lessen the potential for experiencing food insecurity. The interview guide was pretested among the research team and minor revisions were made to improve flow and clarity.

### Data collection

The eligibility screener was sent to mothers via an electronic link. All interviews were conducted by the first author via Zoom. The first author received training to conduct the interviews from PhD-trained researchers with experience using qualitative methodologies (SM and KP). Virtual interviews allow broader geographic recruitment and promote participant comfort because they can choose the location [47]. All participants elected to use the video feature during their interview, although 2 participants turned the feature off to improve internet connection stability. Interviews lasted on average 37 min (range: 20–60 min). Only the participant and interviewer were present for the interview. All interviews were audio-recorded and the interviewer took notes during the session.

### Analysis

The first author reviewed her notes after each interview and noted key concepts. After every 4 interviews, she created memos about emerging concepts across interviews. Data saturation was reached after 15 interviews when no new concepts emerged in 2 consecutive interviews based on the memos [48].

Audio recordings were transcribed by Zoom software and cleaned by the first author. A modified rapid coding technique was used to identify emerging themes because this technique has been shown to produce comparable findings to traditional coding in less time [49]. The first author created structured summaries for each transcript and 2 coauthors (KP and SM) summarized 7 randomly selected transcripts to minimize definitional drifting, where the definition of a code unintentionally strays from the original definition. Agreement between coauthors was >90% on these summaries. Next, 3 authors involved in coding (RL, KP, and SM) independently reviewed the summaries and identified potential themes. They met to compile and condense the independently identified themes. This process led to the identification of the 3 themes presented in the results. The first author then reviewed each transcript and identified passages relevant to each theme. Next, she and another coder (KP) reviewed and organized passages into subthemes. The third coder (SM) reviewed the final themes and subthemes.

## Results

Mothers (*n* = 15) predominately identified as White and not Hispanic or Latino (80.0%, *n* = 12), lived in a household experiencing very low food security (66.6%, *n* = 10), and reported a score of 48.8 (range 25.8–64.6) on the PROMIS Global Mental 2a Scale., with a higher score indicating better mental health and a score of 50.0 being average for the United States population. Participant demographics are presented in Table 1.

**TABLE 1:** Participant race, food security status, and overall mental health score among mothers in Virginia with experience of food insecurity in the past 12 mo

Participant	Race and ethnicity	Food security status^1^	Overall mental health score^2^
1	White	High/marginal	44.4
2	White	Very low	25.8
3	White	Very low	40.6
4	White	Very low	36.5
5	Black or African American	High/marginal	64.6
6	White	High/marginal	44.4
7	White	Low	57.7
8	White	Very low	52.8
9	White	Very low	36.5
10	White	Very low	57.7
11	White	Very low	57.7
12	White	Very low	44.4
13	Black or African American	Very low	52.8
14	White	Very low	57.7
15	Black or African American and Hispanic	Incomplete screener	57.7

1 Food security status was assessed using a 6-item USDA Household Food Security Module Short Form. People were categorized as high/marginal (0 and 1 affirmative responses), low (2–4), or very low (5 and 6) food security based on score thresholds outlined in the implementation guide [45].

2 Overall mental health was assessed using the Patient Reported Outcome Measures Information System (PROMIS) Global Mental 2a scale. Raw scores were converted to T-scores standardized to the United States population with an average score of 50 and 10 points indicating one standard deviation. Higher scores are indicative of better overall mental health. Scores on this scale can range from 25.8 to 64.6.

The 3 emergent themes were as follows: *1*) stress from experiencing food insecurity manifests in multiple ways, *2*) mothers engage in multiple coping strategies to alleviate stress caused by food insecurity, and *3*) stresses caused by food insecurity and resulting coping strategies impact mothers’ mental health. Each theme had 3–5 subthemes. Table 2 presents exemplar quotes organized by subtheme, whereas Table 3 presents the distribution of identification of themes and subthemes by the participant.

**TABLE 2:** Supplementary and exemplar quotes related to mental health and food security mentioned among mothers in Virginia with experience of food insecurity in the past 12 mo by subtheme (*n* = 15)

Theme 1: stress from experiencing food insecurity manifests in multiple ways	
Part of larger financial stress	I have to pay all the bills…it’s not an option and then our basic necessities…and then at the bottom I’ve got our debts. They have to be paid off somehow so I pay the bare minimum on that stuff [debt]. (Interviewee 2) Less than $1000 in my bank account and rent’s due in a couple of days, it’s really stressful. (Interviewee 8)
Worry about getting the “right” foods	I had to gripe a little bit about the price [of fresh fruit]. [I]t’s kind of ridiculous but…we still buy them because of the fact that our kids don’t like vegetables, so we don’t want to short them on fruit. (Interviewee 6) Just trying to navigate having a small child, what’s available, what’s healthy…there were many factors that were stressful, so yeah. Not so good feeling. It’s not a good feeling. (Interviewee 9)
Accessing resources adds extra burden	They [Food pantries] need like, proof of income, [but] they won’t accept a bank statement. Then, you have to have like a letter, so you'd have to go to social services and get that. So that takes forever and it's just like not very pleasant. They make it tricky, especially when you have kids you don't want to haul your kids to do all that and then the cost of gas, …. (Interviewee 9) We get food stamps, but we don't… it doesn't really help get us all the way through the month because it's still going off what my husband was making [before he became unemployed several months ago], you know, with both of our incomes [even though we only have the one now]. (Interviewee 4)
Perceived and experienced stigma	“I feel like a crappy parent sometimes when it gets, when it gets to times like that where I’m running low on food and have to count on other people to help me.” (Interviewee 1, internalized social stigma and self-sufficiency stigma) “I should be able to get it [food] on my own [but I am not].” (Interviewee 4, self-sufficiency stigma) Oh, that makes me feel horrible, because I really would rather people not know that I'm struggling … I literally would have to be like on my last last last last last [dollar] before I ask anyone. For me to still be asking my parents for money, it's kind of humiliating … because I feel like they should be asking me for help right now. (Interviewee 13, internalized social stigma) “You see on [social media] all the time people putting other people down because you're not able to do more for your own family.” (Interviewee 14, experienced social stigma)
Theme 2: mothers engage in multiple coping strategies to alleviate stress caused by food insecurity	
Use available social and/or community resources	We’ll post on a Facebook group, and say, ‘hey anybody got some free food?’ and people will pop by and bring like a box of, like, free produce from the pantry. (Interviewee 3, using social media) You can't buy it [special baby formula] in stores, it has to be ordered and their website has been out of stock and its super expensive, like, for four cans it's $167. And four cans last not… not even a month. I just got WIC for her, like four weeks ago, and they were able to get her formula and have it shipped here. (Interviewee 9, social programs/WIC) [M]y husband is the one that goes [to the food pantry] only because he's not working right now and they're only open on Thursdays, from nine to 11. He says, everyone is very friendly and they help out…so I think after a while once he got familiar with the people that work at that the pantry, he wasn't embarrassed anymore and we're just grateful that they're able to bless us in that way. (Interviewee 13, social programs/food pantries)
Engage in actions to alter financial status	We cut the cable off and I bought a [device to connect television to internet] and we got prepaid Internet through [provider] instead of paying [provider] for regular Internet which, I don't know why I didn't think of that sooner. Because that actually saved us a lot of money. (Interviewee 4, reduce expenditures) At the beginning of the month, when there's a lot of benefits, we eat a lot more convenience foods. But then, as it gets closer to the end of the month we're eating a lot more whole foods, because they're cheaper to make meals out of. They’re not necessarily cheaper to obtain but it's gonna go farther than, say, like a bag of chicken nuggets. (Interviewee 14, changing shopping patterns) I’m trying to find a new job that pays better so that we don’t have to worry as much about just the basics and living paycheck-to-paycheck to hopefully lead a little bit of a more stress-free life for us and the kids. (Interviewee 3, changing jobs) I think that it's hard for me and sometimes I have a lot of guilt. Because I don't want my kids to be raised in this type of situation. My 16-year-old has lived her entire life this way and I don't want my four-year-old to also have to live her entire life this way…I’m in [community college] trying to do better, I guess, you could say it motivates me because I want to get through school, so that we don't have to live like that. (Interviewee 14, obtaining additional education)
Reframe shopping expectations	So, it’s [husband’s unemployment] a huge pump the brakes moment. Like, I never realized how much of my paycheck we actually blew on just going to the grocery store and buying all these fancy [products], like all two cases of soda instead of one … because I didn't really have to worry about the groceries so much…losing all his income, kinda, I just hit a wall. (Interviewee 4) We are surviving with the resources that we have and we cannot afford organic produce. We can hardly afford organic baby formula. So you just try to do your best and your kid is going to be okay. (Interviewee 7)
Reduce personal food intake	I always make sure the kids have what they need. So, even if it's just, “oh, you know,” there’s been a couple nights where we've had to just make a small little dinner, and we fed the kids and then whatever was left after we fed the kids we just shared. (Interviewee 4) The pantry was down to little to nothing, so it was like one night, we had enough meat to do for the kids to eat and I ate a can of green beans. (Interviewee 11) Sometimes I don't eat as much as I probably should just so that my kids will have food... So, if they want extras or something else, they can have that. (Interviewee 12)
Implement avoidance behaviors	I try not to go to the food banks, because, at least we have a home, and… there's other people that aren’t so fortunate…(Interviewee 4, avoid supportive resources) My mom came to visit and she opened the fridge and she was like … this milk is basically cheese and this pizza looks like it's been in there for two weeks’ and I was like, ‘yeah mom, but when I open the fridge it looks full - that's why I haven't thrown anything away because it helps to see that full fridge’, because when I see an empty fridge it's like, ‘oh my God I really have no [expletive] food.’ (Interviewee 4, avoid thinking about food insecurity) … I think part of the reason why I don't tell them [her parents] that I skip meals is because I’m too proud to ask for more money. (Interviewee 8, avoid talking about experiences)
Theme 3: stresses caused by food insecurity and resulting coping strategies impact mothers’ mental health	
Reduces bandwidth negatively relates to mental health	It [getting more SNAP benefits at the beginning of the month] will be a little bit, um, lighter on my brain. Being that I pay my bills at the beginning of the month as well, kind of interfering, like, bumping… bumping heads. (Interviewee 5) I do, a kind of a closer inventory of what we have and pay more attention to just consume things more wisely so it's a little more brain power which is fine, I don't mind that part but it's just thinking about it more. And it becomes such a… a bigger part and that… that displaces energy you have for other things. (Interviewee 10) Sometimes, I think that if I were a better planner then it would go better. It's just so hard at the beginning of the month because we've gone such a long time [since the start of the previous month] with really meager amounts and then, when the… the beginning of the month hits, we’re like ‘woohoo.’ (Interviewee 14)
Adds to existing mental health challenges	I have panic attacks, especially on payday… payday is the worst. You think you would get relief on payday but it's the worst. Like I said I got paid yesterday, and I think I'm down to like $75 now… I probably paid … my car insurance, the electricity, [and] I didn’t go food shopping… So, it's constant worrying and… and I don’t know I probably have some other undiagnosed [mental health conditions]. (Interviewee 11) I suffer from depression, too, but that's not necessarily from the food or whatever that's just from, shoo yeah, that's just from everyday life. (Interviewee 12)
Implementing strategies to address food insecurity has negative mental health consequences	[Using coupons] makes me feel savvy for being able to save money in a certain way, but…there's also an element of shame to it, that I have to use that in order to afford all the groceries that I, not even want, but the groceries that I need. (Interviewee 3) Um it's hard, because sometimes we [she and her partner] end up not wanting to be around each other, because we've been arguing. And sometimes the argument may not even be over food, but it's worse because we're both hungry and we're tired… (Interviewee 14)

SNAP, Supplemental Nutrition Assistance Program; WIC, women, infants, and children.

**TABLE 3:**
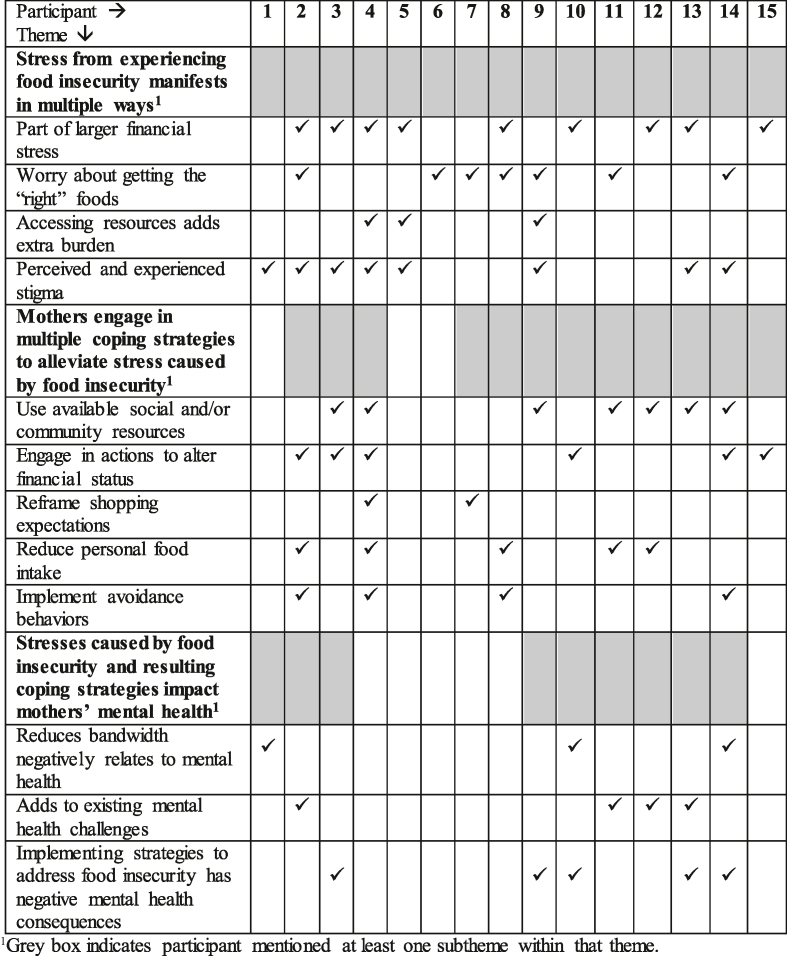
Distribution of themes and subthemes across participants (*n* = 15)

### Theme 1: stress from experiencing food insecurity manifests in multiple ways

Mothers identified multiple ways food insecurity added stress to their lives. More than half of mothers identified 2 or more different stressors.

#### Part of larger financial stress

Many mothers discussed the stress from food insecurity in the context of all the other financial responsibilities they managed. These other responsibilities included mortgage/rent, bills, transportation, childcare, and debt payment. Ensuring that there was enough food in the household was intrinsically linked to these other responsibilities, because mothers often had to make decisions about which responsibilities were prioritized due to limited financial resources. For example, Interviewee 13 discussed her experience of having to decide which bills to pay:

Food is a priority, obviously, just, you know when you have kids, but at the same time, … in January we didn't have any heat because my gas was cut off… And, I think I had to pay whatever amount, so I had to decide, am I going to get my gas turned back on or am I going to buy food? And I had to turn the gas back on because it was so cold outside.

Many participants talked about this stress in the additional context of living paycheck-to-paycheck. They discussed how a large bill or unexpected need can lead to complications with balancing budgets. Interviewee 3 described this when she said,

We're kind of paycheck-to-paycheck right now, there's some weeks where we're fine, but when big bills happen or something unexpected pops up, then it starts to get really difficult to feed everyone, and to feed everyone in a healthy way, which is what I try to do.

Several participants also mentioned the rising costs of gas and food.

#### Worry about getting the “right” foods

Participants discussed their inability to get the foods they wanted for their households. They specifically talked about obtaining nutritious foods and/or the foods their children preferred. Interviewee 14 directly spoke to this theme when she felt “frustrated at not being able to have the meals that I want to have for the kids, or, you know, not being able to provide what I think that they should have throughout the day.”

Recommendations by medical professionals and government agencies that provide food benefits on child feeding practices added to the frustration mothers felt at their inability to obtain the healthy foods they wanted for their children. Interviewee 6 stated her frustration:

They're [the government] always talking about “nutrition is key for families” and… the food pyramid that they say, is so important, then make it inexpensive so that families don't question a bag of chips versus a bag of apples, you know.

#### Accessing resources adds extra burden

Although federal assistance programs [e.g., Supplemental Nutrition Assistance Program (SNAP) and the Special Supplemental Nutrition Program for Women, Infants, and Children (WIC)] and food pantries provide resources that could alleviate food insecurity-related stress, mothers reported the process of accessing these as a stressor. Interviewee 9 described this when she said:

They [Food pantries] need like, proof of income, [but] they won’t accept a bank statement. Then, you have to have like a letter, so you'd have to go to social services and get that. So that takes forever and it's just like not very pleasant. They make it tricky, especially when you have kids you don't want to haul your kids to do all that and then the cost of gas, ….

Additionally, participants noted after they were enrolled in nutrition assistance programs, the system was slow to adjust to changes in income. For example, several months after her husband became unemployed and the family submitted this change, Interviewee 4 noted:

We get food stamps, but we don't… it doesn't really help get us all the way through the month because it's still going off what my husband was making, you know, with both of our incomes [even though we only have the one now]

#### Perceived and experienced stigma

Participants also discussed feeling social and self-sufficiency stigmas. Social stigma was anticipated (perceived), internalized, and enacted (directly experienced). Related to anticipated stigma, parents discussed 2 types: bad parent and self-sufficiency. Bad parent stigma was associated with internalized guilt over not being able to meet their children’s needs and wants and, when their children were aware of the household’s food insecurity, causing their children to worry. Interviewee 8 described her experience with this internalized stigma:

The worst part is if the kids really want, like, apple juice or something, … and like, I couldn't buy it. It’s the worst feeling as a parent [that] … you can't get your kids toys and stuff.

Self-sufficiency stigma was related to perceived expectations that parents should be able to provide for their children without assistance and that because they could not they were not doing what they were supposed to. In addition to their own internalized and anticipated stigmas, mothers also reported directly experiencing judgment and enacted stigma from others. Notably, mothers, such as Interviewee 9, reported experiencing stigma at food pantries:

I don't like to go [to the food pantry] … you feel judged by the workers and the other people there. One time I went and some lady… I don't think she worked there, I'm not really sure who she was, made a comment because I had my nails done, and she was like oh well, ‘why are you here if, like, you can afford your nails done?’ [Getting my nails done] was a birthday present so that was like… not a very good experience.

### Theme 2: mothers engage in multiple coping strategies to alleviate stress caused by food insecurity

Mothers discussed 5 coping strategies they used to reduce stress caused by and/or cope with food insecurity. More than half of mothers who described coping strategies identified using multiple strategies.

#### Use available social and/or community resources

Mothers mentioned using social programs, including SNAP, WIC, and food pantries. They also discussed seeking out support from family members and friends. Interviewee 11 described getting food from her mother when she was experiencing limited resources to help “get us through [a food shortage].” Mothers also reported finding various sources of support through the larger community. For example, Interviewee 3 was part of a community Facebook group where people shared food: “we’ll post on a Facebook group, and say, ‘hey anybody got some free food?’ and people will pop by and bring like a box of, like, free produce from the pantry.”

#### Engage in actions to alter financial status

Participants discussed taking actions to alter their financial status as a means to reduce their food insecurity, such as reducing spending on food and other expenses. Changes in how they purchased food included shopping sales, using coupons, switching to less expensive stores, buying in bulk, and buying cheaper alternatives. Although some mothers reported using these strategies regularly, others used them only when needed. Interviewee 14 described how her family’s shopping habits and resulting consumption changed over the course of a month:

At the beginning of the month, when there's a lot of benefits, we eat a lot more convenience foods. But then, as it gets closer to the end of the month we're eating a lot more whole foods, because they're cheaper to make meals out of. They’re not necessarily cheaper to obtain but it's gonna go farther than, say, like a bag of chicken nuggets.

Mothers also described reducing spending beyond food by cutting cable, reducing internet costs, and conserving electricity. Interviewee 4 described the changes her family made:

We cut the cable off and I bought a [device to connect television to internet] and we got prepaid Internet through [provider] instead of paying [provider] for regular Internet which, I don't know why I didn't think of that sooner. Because that actually saved us a lot of money.

Also, some mothers discussed seeking additional employment, seeking a new job, or furthering their education (Table 3). Similarly, some mothers also described taking on additional employment when resources were constrained (Interviewee 15).

When my son is visiting his father, I have a little free time. I will go [drive for a food delivery service] and of course that's based off of like what my [bank] account looks like. If I’m like ‘oh, we’re kind of low on funds’ and I have the time then I’m going to go out and [drive for a food delivery service]. I don’t like it to be honest…In the limited time that I do have for myself, I don’t want to be [driving for a food delivery service].

#### Reframe shopping expectations

Several mothers reported reframing their expectations for how they viewed ways to shop for food to provide to their family. For some mothers, they started to consider behavioral food coping strategies while shopping, when that had not previously been part of their shopping experience. Interviewee 4 described this when she said:

So, it’s [husband’s unemployment] a huge pump the brakes moment. Like, I never realized how much of my paycheck we actually blew on just going to the grocery store and buying all these fancy [products], like all two cases of soda instead of one … because I didn't really have to worry about the groceries so much…losing all his income, kinda, I just hit a wall.

Other mothers reframed their goal from striving to provide healthy foods to just getting enough of the kinds of foods their family would eat. Interviewee 7 described “surviving with the resources that we have and we cannot afford organic produce. We can hardly afford organic baby formula. So you just try to do your best and your kid is going to be okay.”

#### Reduce personal food intake

Some mothers reported reducing their food consumption to ensure that their children had enough food. As Interviewee 2 highlighted, mothers put their children’s needs ahead of their own: “I won’t let them go hungry. I’ll do without before I let them.” Interviewee 8, who reported consistently reducing her consumption multiple times a week, described her experience:

I mean they’d [the government] come get my kids from me, [so] they've got to be able to eat. But I can be hungry.; They're not going to come put me in jail for questionable diet habits, you know what I mean? … So it's not super healthy, and it's not ideal, but it's not hurting anybody, but me.

Among mothers who reported reducing personal food intake, some reported eating smaller portions or leftovers from their children’s plates and some reported skipping meals entirely.

#### Implement avoidance behaviors

Mothers also reported using avoidance behaviors that allowed them to hide their food insecurity from themselves or others. Interviewee 8 described not asking her parents for help:

When I have these issues [food shortages], I’m not very vocal about like the hunger thing, right? I'll talk to family about everything else. But that's one that I don't really, because the kids are doing all right, and like I’m also like making it.

Also, some mothers, such as Interviewee 4, reported coping by trying to avoid thinking about their food insecurity.

My mom came to visit and she opened the fridge and she was like, ‘[Interviewee 4], this milk is basically cheese and this pizza looks like it's been in there for two weeks’ and I was like, ‘yeah mom, but when I open the fridge it looks full - that's why I haven't thrown anything away because it helps to see that full fridge’, because when I see an empty fridge it's like, ‘oh my God I really have no [expletive] food.’

Lastly, many mothers avoided using supportive resources, especially food pantries, because they did not or could not identify as someone who used those types of resources. Similar to other mothers, Interviewee 2, described not using food pantries because those are “for people that really, really, really need it.”

### Theme 3: stresses caused by food insecurity and resulting coping strategies impact mothers’ mental health

Three ways stress and coping strategies impacted maternal mental health were identified: adding to existing mental health issues, limiting bandwidth (mental resources) to change, and strain from the effort to conserve resources.

#### Reduced bandwidth negatively relates to mental health

Mothers reported a general sense of worry associated with food insecurity. Interviewee 1 described how reducing her food insecurity would make her feel: “It [Reduced food insecurity] would be a big relief off my back like I wouldn’t have to worry so much all the time about [getting food].” This worry, along with all the competing stressors in their lives and their efforts to cope with them, left some mothers with limited bandwidth to make changes. Some mothers recognized that there were things they could do to improve their circumstances, but they were so overburdened managing their situation that they did not have the capacity to implement change. Interviewee 14 described her challenge with improving her food resource management:

Sometimes, I think that if I were a better planner then it would go better. It's just so hard at the beginning of the month because we've gone such a long time [since the start of the previous month] with really meager amounts and then, when the… the beginning of the month hits, we’re like ‘woohoo.’

#### Adds to existing mental health challenges

Some mothers reported managing diagnosed mental illnesses or mental health challenges. Many of these mothers, such as Interviewee 12, attributed their mental illness to other causes: “I suffer from depression, too, but that's not necessarily from the food or whatever that's just from, shoo yeah, that's just from everyday life.” Some mothers directly acknowledged food insecurity, and stressors associated with it, and were exacerbating their symptoms. Interviewee 2 shared how food insecurity worsened her mental health: “[diagnosed] anxiety, [diagnosed] depression, frustration… which I already have a lot of these issues, due to my disability but this [food insecurity] just adds to making the mental health worse.” Lastly, Interviewee 13 highlighted how the financial stress mothers are managing both exacerbate symptoms of an existing mental illness and contribute to generally poor mental health outcomes:

I have panic attacks, especially on payday… payday is the worst. You think you would get relief on payday but it's the worst. Like I said I got paid yesterday, and I think I'm down to like $75 now… I probably paid … my car insurance, the electricity, [and] I didn’t go food shopping… So, it's constant worrying and… and I don’t know I probably have some other undiagnosed [mental health conditions].

#### Implementing strategies to address food insecurity has negative mental health consequences

Finding strategies to stretch limited resources caused some mothers to experience poor mental health outcomes. For some mothers, such as Interviewee 3, there was a conflict between feeling creative and embarrassed about using strategies to save money:

[Using coupons] makes me feel savvy for being able to save money in a certain way, but…there's also an element of shame to it, that I have to use that in order to afford all the groceries that I, not even want, but the groceries that I need.

Interviewee 13 also experienced conflict between savviness and shame: “I know I’m doing the best that I can, but it still makes me feel bad just because, I don’t know, it makes me feel poor to be honest with you.”

The many stressors that mothers manage also strained relationships. Interviewee 14 described the impact of these strains on her relationship with her partner:

Um it's hard, because sometimes we [she and her partner] end up not wanting to be around each other, because we've been arguing. And sometimes the argument may not even be over food, but it's worse because we're both hungry and we're tired…

## Discussion

The purpose of this study was to describe the stressors of mothers experiencing food insecurity, their coping strategies, and their impact on their mental health in the context of the continuing COVID-19 pandemic effects on price inflation and the infant formula shortage [42,43]. The findings of this study in that context were consistent with existing literature suggesting food insecurity adds stress to mothers’ lives in multiple different ways [50,51] and that mothers engage in multiple coping strategies to mitigate that stress [52,53]. The novelty of this study was that mothers identified multiple ways through which stress associated with food insecurity and coping strategies negatively impacted their mental health. This understanding of mothers’ experiences of food insecurity may help identify potential areas to develop interventions designed to reduce food insecurity that align with mothers’ preferred coping strategies.

United States cultural, economic, social, and political systems are largely based on a neoliberal ideology focused on individual/self-determination, personal responsibility, hard work, and economic citizenship [54]. This can translate to social stigma that suggests people living in poverty are not working hard enough and therefore deserving of poverty and food insecurity and, as a result, shame. This ideology also does not recognize the economic landscape or the realities of “the working poor” [54]. Furthermore, the intensive mothering ideal in the United States pressures mothers to strive for perfection and be able to meet their child(ren)’s needs without relying on any form of assistance [55].

Perpetuation of these ideals ignores systemic barriers to food security and contributes to anticipated, internalized, and enacted social stigma that keeps mothers from using needed resources, as seen in this study. This is consistent with significant evidence suggesting people perceive a stigma surrounding SNAP [[56], [57], [58]] and food pantry utilization [[59], [60], [61]]. Food pantries, in particular, have been consistently underutilized by people experiencing food insecurity [[59], [60], [61]]. However, the use of food pantries increased during the COVID-19 pandemic nationally and in specific cities [[62], [63], [64], [65]]. Although more research is needed to explain this trend, support for assistance programs is associated with a belief in systemic, rather than individual, causes of poverty [66]. Acknowledging the role of the COVID-19 pandemic as a systemic factor may have reduced the stigma associated with pantry usage. Because more people refuse to accept the prevailing neoliberal ideology and utilize needed resources, they contribute to further reducing stigma [67]. Reducing the stigma associated with resource utilization can help improve food security by even moderately increasing the social acceptability of acquiring food through these channels in the short term and may lead to increased support for systemic changes in the long term.

In the absence of a clear path to restructuring the systems that perpetuate inequities contributing to food insecurity, efforts to assist mothers already coping with food insecurity may mitigate some of the burden. Resilience, which is how household assets and resources interact with a crisis, can explain why households may experience disparate outcomes [68]. Understanding the assets and challenges of a given household that contribute to resilience may help explain both the experience of food insecurity and the impact on the mental health of mothers [58]. Participants demonstrated resilience through some of their coping strategies (e.g. engaging in actions to alter financial status, using available resources, and reframing shopping expectations). Although successfully managing a previous crisis can strengthen resilience to future challenges, consistently managing crises can impact mental health and reduce future resilience [69]. Systemic changes are needed to help households avoid consistent crises.

The breadth of stressors mothers experienced was consistent with the literature [51]. Participants reported stress associated with balancing financial responsibilities, putting nutritious food on the table, and perceived and experienced stigma [51]. There is some evidence to suggest stress as a pathway through which food insecurity impacts both physical and mental health [34,70,71]. Although the specific pathways need to be further explored, it has been proposed that stress, potentially through neuroendocrine disruptions or cortisol release, may contribute to poor physical health outcomes [34,70,71]. Maternal stress has also been associated with higher weight among children in the household [34].

Given the wide range of stressors felt by mothers experiencing food insecurity and the implications for their health, nutrition education programs targeting participants with low income may need adaptations to fully meet the needs of mothers. Many of these programs, similar to the Expanded Food and Nutrition Education Program, primarily focus on improving food resource management, nutrition, and food security among mothers and caregivers with low incomes [72]. Although these are important concepts, many mothers in this study identified a need for other coping strategies but reported insufficient bandwidth to implement additional strategies, such as monthly budgeting. Furthermore, increased reliance on behavioral food coping strategies has previously been associated with poor mental health outcomes [5,6]. Future research should explore effective strategies for addressing stressors and methods for incorporating those into existing nutrition education programs without perpetuating self-sufficiency stigma. Additionally, efforts to shift the paradigm to dismantle both neoliberal and intensive mothering ideologies may help reduce the social stigma.

The findings of this study suggest that mothers use multiple coping strategies to alleviate the stress of food insecurity. This is consistent with existing literature suggesting households often have to rely on multiple strategies, especially when household resources are unstable, which may have been the case in this study given the economic context described earlier [52,53]. Although Leung et al. [51] noted primarily negative coping strategies and suggested parents were engaging in emotion-focused coping, mothers in this study reported a range of strategies that were positive (seeking support from social and/or community sources), negative (reducing personal food intake), and potentially positive or negative (engaging in action to alter financial status). Mothers in this study were engaging in all forms of coping (task-, emotion-, and avoidance-oriented). Avoidance-oriented strategies, which unlike task- and emotion-oriented strategies are characterized as not attempting to change the situation [73], are associated with higher stress and increased likelihood of developing depressive symptoms [74]. Future research should explore positive task- or emotion-oriented strategies to help mothers cope with the high burden and lack of real and perceived control associated with managing food insecurity.

There were 3 limitations of the current study. First, participants were aware that the study focused on food insecurity and mental health; therefore, there may have been a self-selection bias. Second, participants were primarily White; therefore, the experience of mothers of color, who may experience different stressors and/or the identified stressors in different ways, was not fully captured. Third, participants needed to have adequate technology access to participate in the virtual interviews [47]; therefore, the experiences of mothers without access were not captured. Lastly, only participants in Virginia were included in the study. Qualitative research inherently incorporates the environment in which the data was collected [75], and therefore, the generalizability of these findings may be limited. Despite these limitations, the themes identified in this exploratory study suggest important parts of mothers’ experiences that should be further explored in future research in more diverse populations and geographic areas.

Although there is certainly a pressing need for action to address the underlying systemic causes of food insecurity at the community and policy levels, mothers who are utilizing nutrition education programming may benefit from limited, but easily implementable in the short term, changes to improve these programs. Given the many ways that mothers reported food insecurity impacted their lives, a “one size fits all” approach may not be effective. Reimagining nutrition education programs to address multiple stressors impacting mothers, rather than focusing on coping strategies (i.e., food resource management behaviors) without acknowledging stressors, may improve the effectiveness of programs. Additionally, nutrition assistance programs may need to examine how their services contribute to self-sufficiency stigma and identify ways to mitigate it, so mothers feel empowered to use all available resources. Lastly, practitioners should recognize that mothers are willing and capable of juggling competing financial interests and using coping strategies to care for their families, but this places a significant burden on mothers. Incorporating strategies to improve mental health into interventions may be one way to reduce the burden and free some mental capacity to consider the adoption of positive coping strategies.

## Author contributions

The authors’ responsibilities were as follows – RAL, KJP, LMA, VEH, ELS, NEC, SAM: designed research; RAL, SAM: conducted research; KJP, SAM: developed methodology; RAL, KJP, SAM: analyzed the data; RAL: wrote the paper; KJP, LMA, VEH, ELS, NEC, SAM: reviewed and edited the paper; SAM: administered the project and was primarily responsible for the final content; and all authors: read and approved the final manuscript.

## Conflict of interest

The authors report no conflicts of interest.

## Funding

This research was funded by a College of Agriculture and Life Sciences Seed Grant, Virginia Tech. The funder had no role in the design, implementation, analysis, or interpretation of the data.

## Data availability

Deidentified data described in the manuscript, code book, and analytic code will be made available upon request pending approval.
