# Supplementary material for: “I’m Doing the Best that I Can”: Mothers Lived Experience with Food Insecurity, Coping Strategies, and Mental Health Implications
Source: Curr Dev Nutr. 2024 Mar 21;8(4):102136. doi: 10.1016/j.cdnut.2024.102136 (PMC11031732; doi:10.1016/j.cdnut.2024.102136)
Supplement: Multimedia component 1 [file mmc1.docx]

**Supplementary Table 1: Mental Health and Food Security Interview Questions**

| Question 1 | 1. First, I would like to hear about your family’s usual food experiences?    1. What kinds of foods you and your family eat?    2. How you usually get food?    3. Who usually buys the food?    4. How often do you buy food? |
| --- | --- |
| Question 2 | I would like to know more about what you do when you worry about or struggle to get enough of the kinds of foods that you would like to get for your household.  When has this happened before? *Probe for on-going and one-time causes*  What (if anything) did (do) you/your family do to reduce your worry or to get food into the household? *After the initial answer, probe to get them to explain their strategies, why they chose/used that strategy, how they made the strategy work, and how using the strategies made them feel. Also, probe to identify all strategies that they used (physical, mental, social network for financial/emotional/material support*) |
| Question 3 | 1. How did these experiences worrying about or working to get enough or the kinds of food that you would like for you household impact your mental health?    - 1. Did they cause anxiety or depression?      2. What things, if any, helped your mental health during these times? *Probe into impact of the specific strategies mentioned.*      3. What things, if any, worsened your mental health during these times? *Probe into impact of the specific strategies mentioned.* |
| Question 4 | 1. When you think about the times you have worried about or struggled to get enough food or the kinds of food that you would like for you household to have, what things could reduce your worry or your struggles? *What would need to happen? Who would need to play a role? How would this impact your mental health?* |
| Question 5 | 1. That was my last questions. Is there anything else you’d like to share about your experience working to get enough or the kinds of food that you would like for your household? |
